# Research highlight: understanding multilevel barriers to childhood vaccination uptake among Internally Displaced Populations (IDPs) in Mogadishu, Somalia: a qualitative study

**DOI:** 10.1186/s12889-024-18102-y

**Published:** 2024-03-18

Vaccines, which save millions of lives annually, are not easily accessible in certain parts of the world, including Africa, with Somalia being especially affected due to political strife and natural catastrophes.

This study aimed to identify obstacles to childhood immunization in Internally Displaced Persons (IDP) camps in Mogadishu. IDP are individuals who have been forced to flee their homes but remain within their country's borders.

This study is a qualitative process evaluation of a cluster-randomized controlled trial that evaluated whether participatory learning and action (PLA) intervention could improve vaccination uptake among children living in IDP camps. The intervention was successful in improving maternal knowledge and vaccination coverage but unsuccessful in improving timely vaccination. The objective of the study was to understand this result and analyze the multi-level barriers to routine childhood vaccination uptake.

The study found that multiple factors, ranging from individual to policy levels, affected vaccination uptake. At the personal level, lack of awareness about vaccine types and schedules, fear of side effects, and opportunity costs were notable obstacles. At the interpersonal level, women often couldn't make decisions regarding their child's health without their husbands' or mothers-in-law's support. At the community level, cultural and traditional beliefs often obstructed vaccination efforts. At the health system and policy level, vaccine supply, human resources, infrastructure, and policy discrepancies between the Somali Expanded Program on Immunization (EPI) and health worker practice emerged as significant barriers.

The research concluded that complex and interrelated factors affect childhood vaccination uptake among IDPs in Somalia. It is crucial to address social, health system and policy barriers. Recommendations included relaxing the age limit for catch-up vaccination in line with the WHO guidelines, implementing interventions that engage families and communities, and addressing reasons why the EPI policy isn’t fully implemented.

*The following summaries of hand-selected papers were generated by Springer Nature's artificial intelligence tool and revised by a subject matter expert to meet Springer Nature's standards*.

